# Prevalence and management practices of ophthalmic lesions in laboratory mice

**DOI:** 10.1038/s41598-026-43181-9

**Published:** 2026-03-11

**Authors:** Dana Matzek, Sonja Rumpel, Stefan Kassumeh, Andreas Ohlmann, Thomas H. Brill, Bastian Popper

**Affiliations:** 1 Core Facility Animal Models, Ludwig-Maximilians-University, Biomedical Center, Medical Faculty, Großhaderner Straße 9 Martinsried, 82152 Munich, Germany; 2 Institute of Animal Welfare, Animal Behaviour and Laboratory Animal Science, Freie Universität Berlin, Berlin, Germany; 3 Department of Ophthalmology, LMU University Hospital, Ludwig-Maximilians-University, Munich, Germany

**Keywords:** Eye, Cornea, Rodents, Animal welfare, Diseases, Health care, Medical research

## Abstract

The regular inspection of the eye health is an essential part of the veterinary care task in an animal facility in order to achieve robust results during experimentation. The aim of the study was to find out, when and how ocular diseases are recorded in laboratory animal facilities and what measures are taken. An online questionnaire was sent to people who work in a research environment and are involved in monitoring of animal wellbeing. In addition, an evaluation of 142 veterinary clinical cases in mice were carried out under routine conditions with the help of ophthalmological tools. 128 participants from three European countries took part in the survey. 93% stated, that eye changes, if recognized, are recorded during cage changing but in 70% of the responses, there was no systematic analysis of the underlying causes. The population study showed that technical aids are indispensable for the detection of eye lesions in mice. The survey and the population study show that although recommended, routine checks of the eye are rarely carried out and usually without suitable technical aids. From the point of view of good veterinary practice, more attention should be paid to the ocular health in laboratory animal husbandry.

**Supplementary Information:**

The online version contains supplementary material available at 10.1038/s41598-026-43181-9.

## Introduction

Laboratory mice (*Mus musculus*) are indispensable models in translational research, offering insights into the genetic, developmental, and environmental factors influencing ocular health^1,2^ With the help of high-throughput methods, multiphoton imaging and omics techniques, a large number of genes, proteins and lipids have been identified in the mouse eye that might be important to mimic ocular diseases in translational research^3,4^. Further, mouse phenotyping and cross-species gene ontology analysis revealed a broad range of genes e.g., in the cornea, that could be potentially drug targets^5^.

Impairments in visual function can confound behavioral data, particularly in studies involving spatial navigation in neuroscience research^6^. From an animal welfare perspective, it is essential to distinguish “acquired”, veterinary clinically relevant lesions from so-called “background lesions” of the species used in order to be able to carry out adequate health evaluation in the best possible way according to the 3Rs principle^7,8^. Background lesions are changes that can be caused by e.g., spontaneous, subline creation of wildtype strains or genetic modification of individuals and are typically attributed as characteristics to a laboratory strain or species and are not limited to one organ system^7,9^. Background lesions covering the ocular apparatus are e.g., cornea opacity, cataract formation, retina degeneration or microphthalmia which have been well documented in rodents^10–15^. Recent research has highlighted the functional sophistication of the mouse visual system and its relevance in natural behaviors like conspicuous recognition^16^. Mice possess dichromatic vision with sensitivity to ultraviolet and green wavelengths having a visual field which is nearly panoramic, stretching from behind the head to below the snout^17^. While some wild-type mouse lines are classified as blind per se due to genetic defects that cause retinal degeneration in these animals and thus affecting the posterior eye segment^9^, there are a variety of changes in the ocular apparatus that can affect the anterior segment comprising the cornea to the lens. These changes range from the background lesions described above to changes caused by the experimental procedures e.g., anesthesia-induced cornea lesions and cataract formation^18,19^ or housing conditions e.g., intra-cage ammonia levels leading to cornea opacity^15^as well as aging-associated factors acting on the cornea architecture^20,21^. Although there are criteria by which deviations from the animals’ physiological condition can be assessed (e.g., the Mouse Grimace Scale^22^,, or the body condition score^23^) assigning symptoms to respective severity categories requires careful evaluation. To facilitate a common understanding, and avoid decisions on a subjective basis, guidelines are available on how experimental interventions could be classified^24–26^. Further, as proposed by the FELASA working group almost all parts of the anterior eye segment should be investigated as part of routine health checks^27^..

In this study, we wanted to evaluate current practices in examination, documentation and management of ocular health during husbandry and breeding in laboratory animal facilities. We evaluated a questionnaire answered by 128 people around Germany, Austria and Switzerland in which we asked for commonly observed eye abnormalities and related procedures to confirm diagnosis under routine situations. Furthermore, we underline the importance of a standardized routine eye examination technique of the anterior segment in the management of laboratory mice by analyzing 142 cases from our facility.

## Results

### Questionnaire

One of the central questions of the study was to find out how ocular abnormalities in laboratory mice are recorded and evaluated in routine laboratory animal husbandry. 128 participants from three German-speaking countries took part in the survey (Supplementary Table S2). Veterinarians (47%) followed by animal technicians (41%) made up the largest number of participants.

The vast majority of participants stated that eye changes were never appreciated (41.53%) or only in individual cases (39.83%) further investigated using special examination methods. Of interest, 60 respondents skipped the question on further diagnostics of ocular lesions. Of the 58 responses, 77.59% stated that sample material or live animals were sent for examination at external institutions. For veterinary clinical diagnostics, 13% of respondents indicated that they had a slit lamp, 3% had intraocular pressure measurement techniques, and 21% had the fluorescein test. We were interested in the frequency with which certain diseases are detected. Lens opacities were diagnosed with 77.59%, followed by 70.69% for microphthalmia. Inflammatory changes of the lid margin (55.17%) and conjunctiva (50%) were more common, as were wounds and injuries to the eyelids (51.72%). Corneal ulcers and oedema of the cornea accounted for 32.76% and 8.62% of cases respectively.

Only 14.41% stated that eye changes were specifically analyzed to determine the causes.

To complete the questionnaire, we wanted to know what causes had been identified so far. This question was answered by 17 participants, most of whom used the terms ‘housing environment’ followed by ‘genetics’ and ‘infections’. As factors that are assumed to be causal, 86 participants answered predominantly with the terms ‘genetics’ followed by ‘housing environment’.

### Ocular investigation

**Fig. 1:**
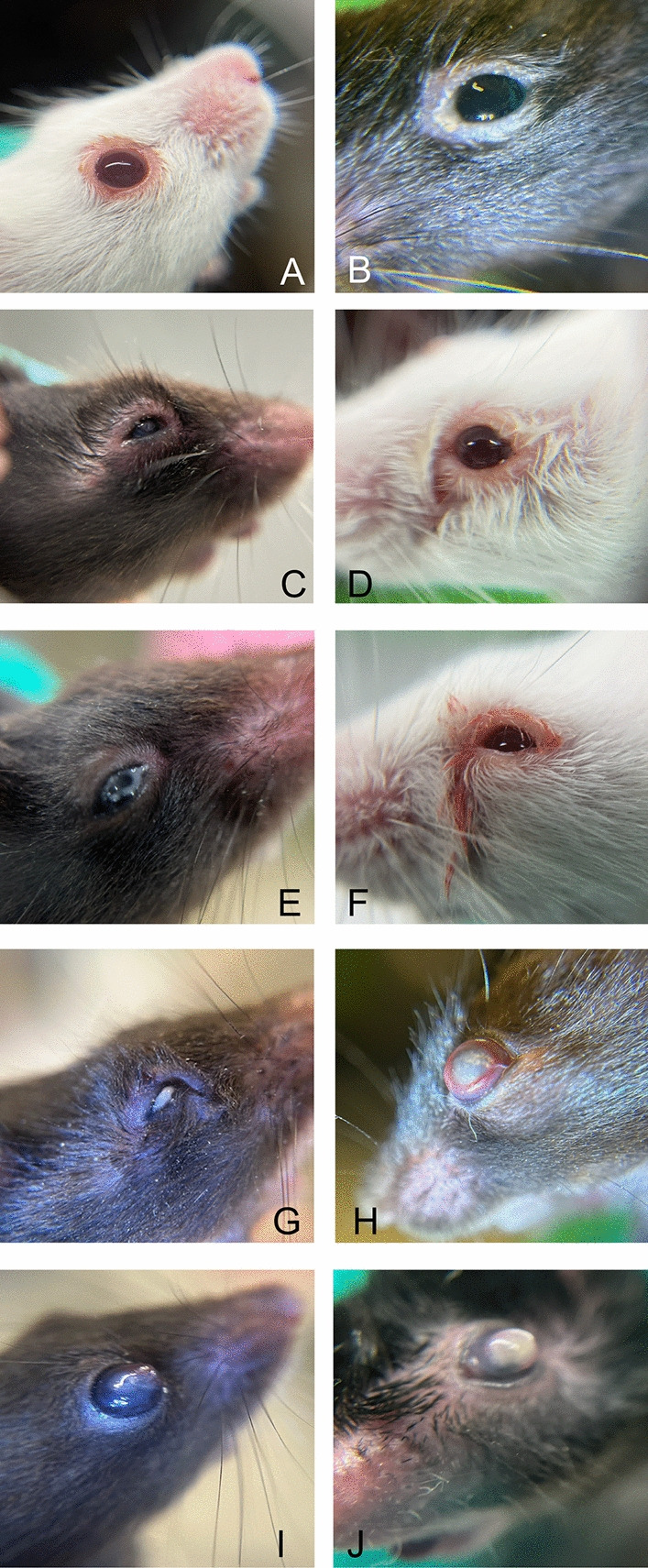
Representative images of eye changes as recorded by staff during routine examinations (**A**)Reddened, slightly swollen eyelid margin with slight exudation (**B**)Hairless eyelid margin with slight swelling and hardly any secretions (**C**) Severely swollen eyelids with altered palpebral fissure, the eyelid margin is reddened and watery secretions adhere to the surrounding hair in the lateral eyelid margin (**D**) Madarosis of eyelid margins (**E**).Moderate swelling of the eyelid with clear yellowish-white exudation and barely visible cornea (**F**).Narrowed palpebral fissures and hemorrhagic discharge of the medial angle of the eye (**G**).Complete displacement of the cornea, including yellowish exudate and massive swelling of the upper eyelid (**H**) Strongly reddened conjunctiva is visible due to the protruding eyeball, the cornea is completely cloudy with a rough surface (**I**) Protruding eyeball with a cloudy cornea and a corneal lesion in the nasal corner of the eye with sprouting superficial blood vessels (**J**) Protruding eyeball with a completely whitish-yellowish color with a shiny surface as well as increased tear exudation and hairless eyelid margins.

Based on ocular abnormalities observed in our facility, we started to systematically record these findings. We investigated 142 cases during a four-month period from October 2024 to February 2025 which accounts for at least 1 case per week within a continuous population of approximately 10,000 animals (Supplementary Figure S1). Ocular abnormalities are either indicated by swollen and hairless eyelids (Fig. 1 A-C) or show discharge of fluids ranging from mucous white to solid yellow and fluid red (Fig. 1 D-G). Other cases show protruding eyeballs (Fig. 1 H-J), whitish discolored cornea (Fig. 1 H-J), and flushed conjunctiva (Fig. 1 H). The predominant number of animals in both cohorts were female mice (GMO and WT), whilst the overall age ranged from 1–31 month (Supplementary Figure S1).

### Cornea

**Fig. 2:**
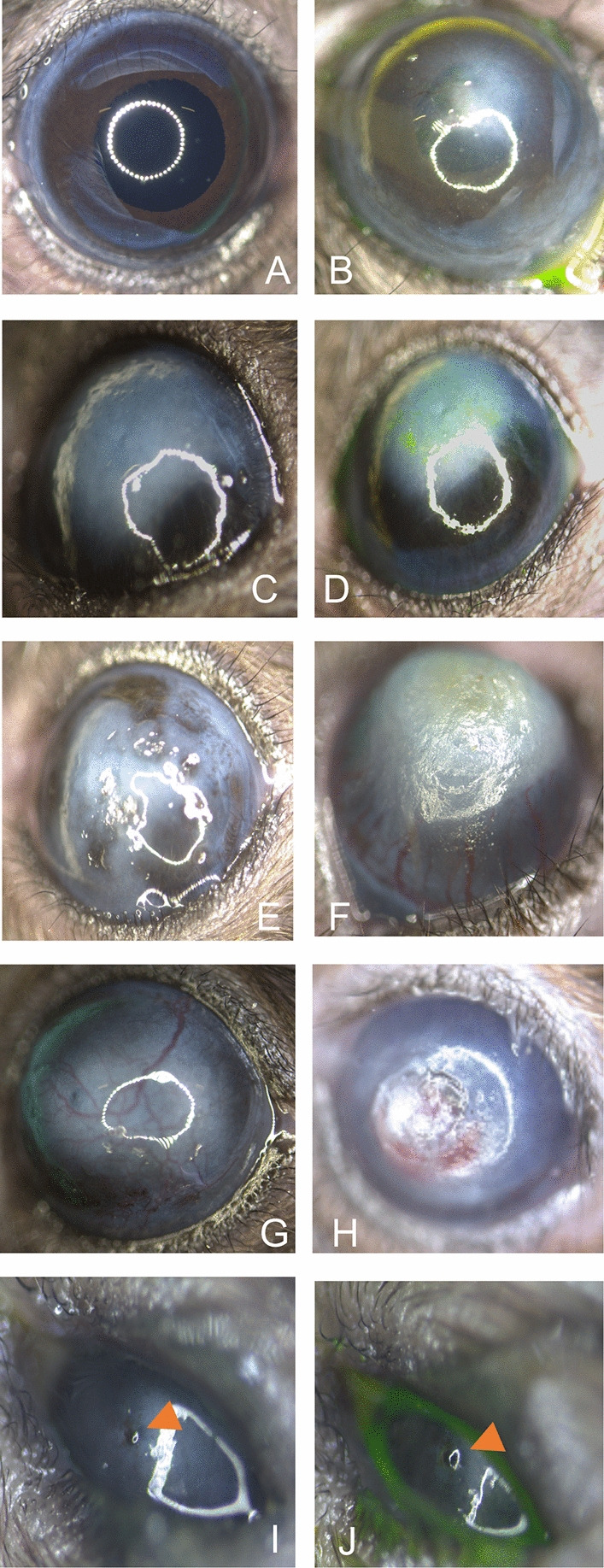
Representative images of corneal lesions (**A**) Representative image of healthy mouse eye showing smooth and shiny cornea and connective membrane (**B**)Irregular opacity of the cornea with scarring (**C**)Central large-area, uniform clouding of the cornea (**D**) Clouding shown in C after fluorescein treatment. Multiple positive staining areas are visible indicating punctate superficial keratitis (**E**)Large corneal scar with incipient superficial vascularization (**F**) Extensive vascularization of the corneal scar from the deep layers of the limbus, ectatic cornea (**G**) Complete corneal opacity with multiple pigmentated areas of different size (**H**) Completely opacified cornea and opening of individual superficial vessels with a central corneal ulcer (**I**) Small but complete cornea ulcer (arrow head) leading to iris prolapse (Descemetocele) (**J**) Negative fluorescein staining conforms complete cornea destruction and iris protrusion.

**Fig. 3:**
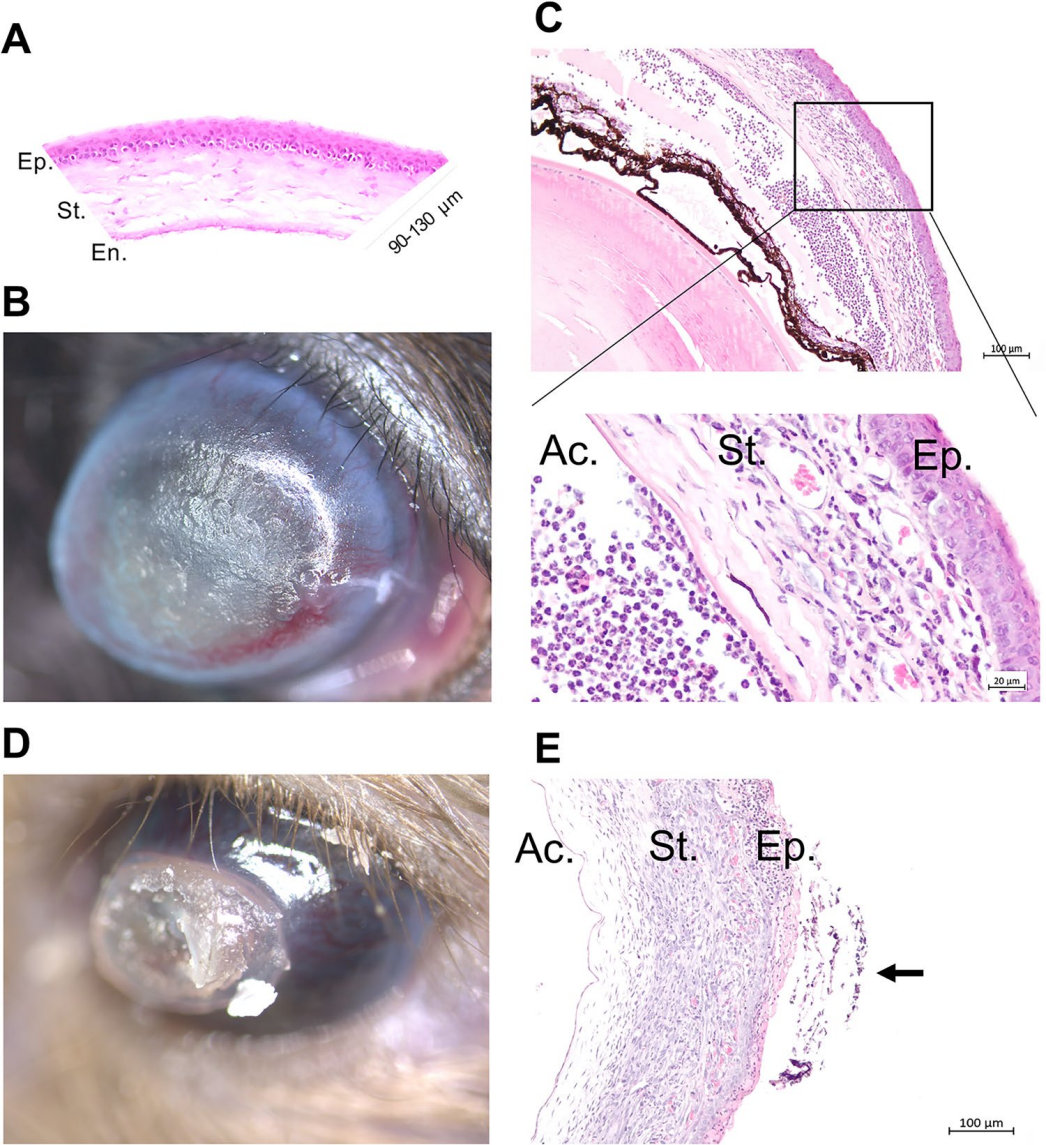
Histological evaluation of corneal lesions (**A**) Representative H&E-stained section of a healthy mouse cornea (**B**) Pronounced keratitis and complete clouding of the cornea with scarring of the surface as well as superficial and deep vascularization starting from the limbus (**C**) Section through the anterior segment of the eye (case shown in B) shows a marked increase in thickness of the cornea and numerous cells (hypopyon) and hyaline material in the anterior chamber. Inset shows neutrophil granulocytes and macrophages in the anterior chamber of the eye plus numerous blood vessels in the corneal stroma, which is heavily interspersed with infiltrating cells (**D**) Corneal opacity and neovascularization as well as central detachment of the upper corneal metaplastic layers (**E**)Section through the cornea segment shows a marked thickening, hyperplasia, subepithelial cellular infiltration, neovascularization and corneal cell detachment (squamous non-neoplastic lesion/arrow) Legend: Ep. = epithelia, En. = Endothelia, St. = stroma, Ac. = anterior chamber.

Whilst 92 cases had a corneal opacity, 18 mice showed cataract, 35 microphthalmic eyes and 16 individuals showed signs of extraocular infection (Supplementary Figure S1). We took pictures to further classify corneal lesions. Starting from a physiological cornea, which shows a smooth and shiny surface and reflects a natural circular reflection of light on the surface (Fig. 2 A), we have recorded a range of corneal opacities (Fig. 2 B-J). The selection shows smaller areas with opacities (B) as well as large-area opacities that respond to fluorescein administration with multifocal fluorescence (C,D) and large-area opacities with pigment deposition (E). In addition, multiple forms of scleral/corneal vascularization can be distinguished (F, G, H). Furthermore, corneal opacities with small punctate defects that do not react to fluorescein administration can be found. In total, 28 cases showed fluorescein staining, whereas 64 cases showed opacities without it. Fluorescein negative opacities were histologically processed for further evaluation. As an example, two cases are shown in Fig. 3 B and D, which showed a different extent of corneal opacity. Extensive thickening of the cornea (C,E) with inflammatory cell infiltration and neovascularization can been seen. Behind the opacified cornea in B, a yellowish-white sheen can be identified, which on histological examination turned out to be purulent anterior uveitis (Fig. 3 C).

### Iris and lens

**Fig. 4:**
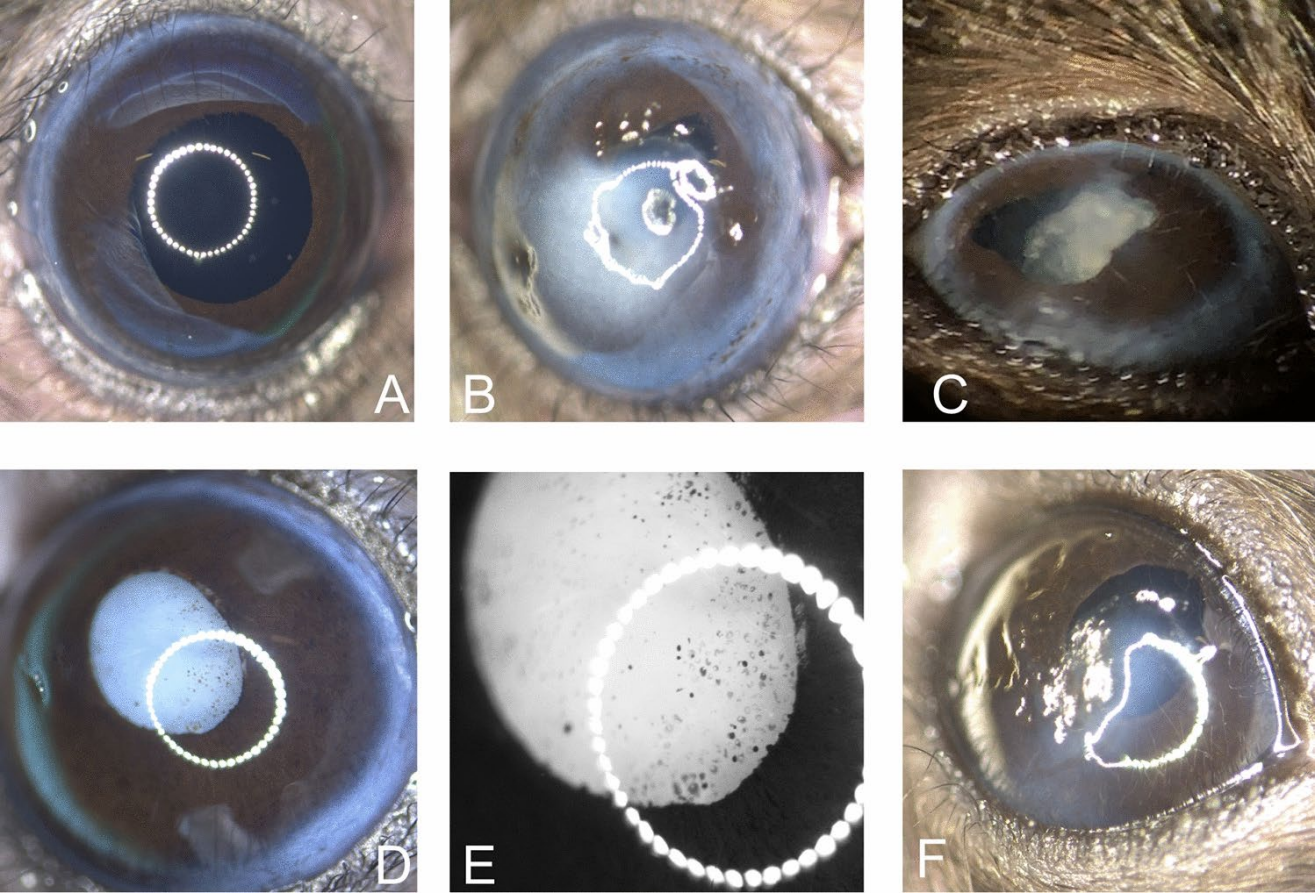
Representative images of deformations affecting the iris (**A**) Representative image of healthy mouse eye showing smooth and shiny cornea and connective membrane (**B**) Elliptical, drawn-out iris margin at the upper edge of the image can be seen behind a central corneal opacity and several pigmentated superficial defects (**C**) Uneven iris margin with otherwise inconspicuous corneal structure. An irregular, cloud-like lens (uveitic cataract) is visible (**D**) Eccentrically located, ovoid, elongated iris shape (posterior synechia) due to adhesion to the completely opacified lens (mature cataract) with otherwise inconspicuous corneal stratification (**E**) Higher magnification of iris margins (picture D) shows pigmentation of the anterior lens (mature cataract) (**F**) Central cornea scar and irregular astigmatism as well as irregular shaped iris margin.

**Fig. 5:**
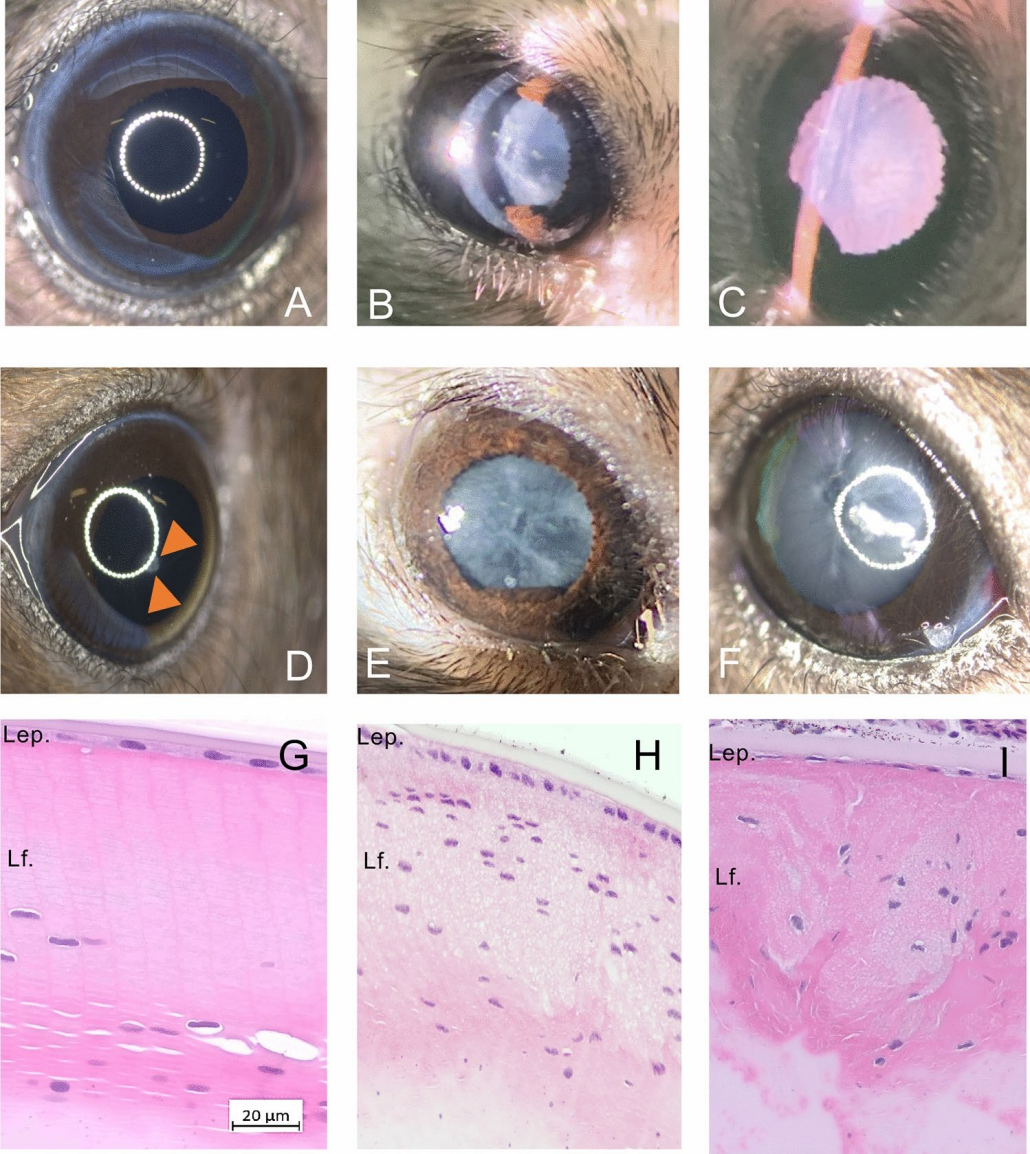
Representative images of lens-associated alterations and histological evaluation (**A**) Representative image of healthy mouse eye showing smooth and shiny cornea and connective membrane. The light passes completely through the uncloudy lens and is reflected by the retina as a black area. (**B**)Slit lamp examination shows light reflections of the lens fibers (**C**) Slit lamp examination shows changes at the bottom edge of the iris that are also easily recognized by the reflection of the lens structure and would not have been immediately noticeable when viewed with a magnifying glass (**D**) Small area with incipient cataract (arrow head) (**E**) Pre-mature cataract (image from C) with opacification of the lens (**F**) Mature cataract (image from C) with even more pronounced lens fiber changes than in E (**G**) Normal architecture of lens fibers (H&E stain) (**H**) Swelling of lens fibers results in altered structure and staining intensity (H&E stain) (**I**) Markedly swollen lens fibers and irregular cellular architecture underneath the lens epithelia cell layer (H&E stain).

Irregular shaped iris that deviated from a physiological rounded structure (Fig. 4 A) or adhesions with the lens could be detected in individual cases (Fig. 4 B-F). Slit lamp examinations (Fig. 5 B,C) showed that in 18 cases there was an opacification of the lens, which was evident to varying degrees and intensity (Fig. 5 D-F) and was accompanied by damage to the cortical lens fibers in the histological evaluation (Fig. 5 H, I).

### Eyelids, glands and fluids

**Fig. 6:**
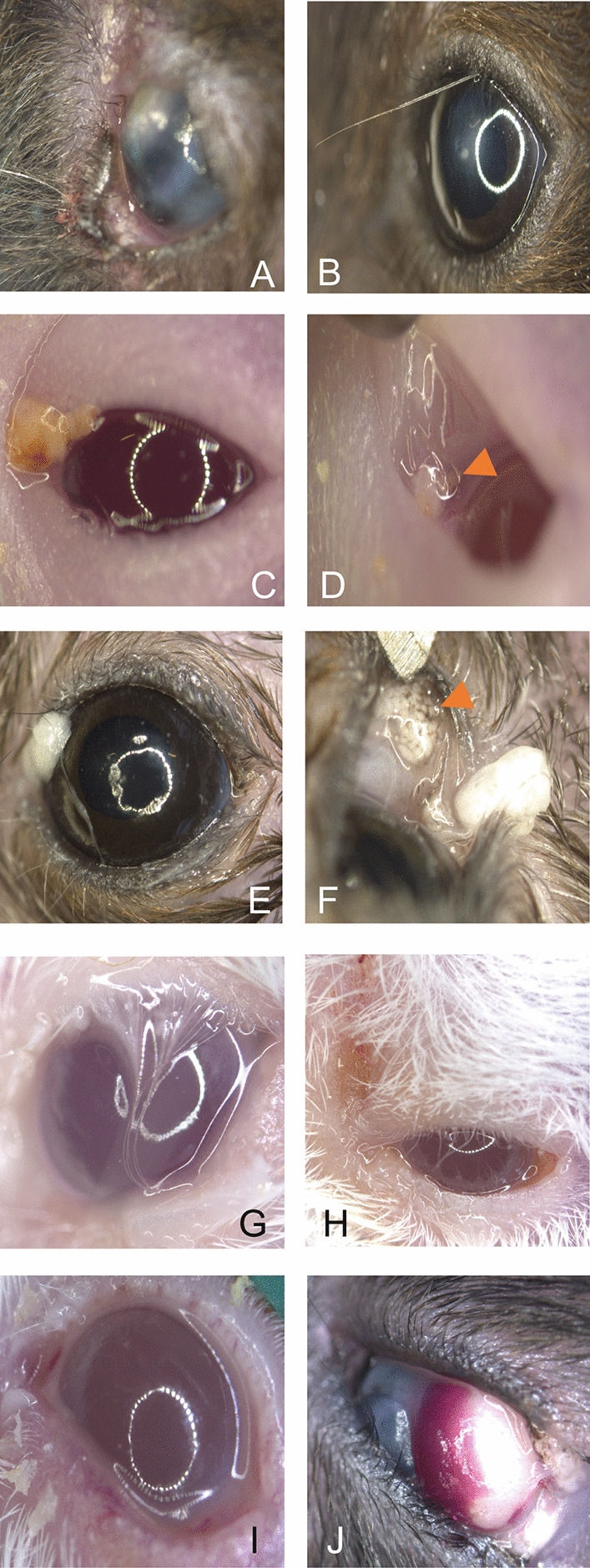
Representative images of eyelids and orbital segment (**A**) Inflammation of the eyelid margin with crust formation. The protruding lower lid margin has secondarily led to a lack of wetting of the eye and caused a cornea ulcer (**B**) A punctured vibrissa in the upper lid margin and central corneal edema (**C**) Eye of a nude mouse with an accumulation of gelatinous-milky secretion (seborrhea) and dust particles in the nasal corner of the eye (**D**) Nude mouse with secretory and dirt concretions in the lower lid margin (arrow head) with mild inflammation characterized by vascular sprouting of the conjunctiva (**E**) Milky, viscous mass (seborrhea) in the nasal corner of the eye as well as sticky eyelashes on the slightly swollen upper and lower lid margin (**F**) View into the lower lid margin shows congested Meibomian glands (arrow head) with otherwise apparently unchanged conjunctiva (**G**) Blepharitis and Seborrhea leading to secondary trichiasis (**H**) Excessive fluid secretion leads to dermatitis in the surrounding eye area (**I**) Blepharitis and conjunctivitis with extensive concrement deposits on the lid margin and nasal corner of the eye in an otherwise irritation-free eye (**J**) Extensive hyposphagma and simultaneous clouding of the cornea.

**Fig. 7:**
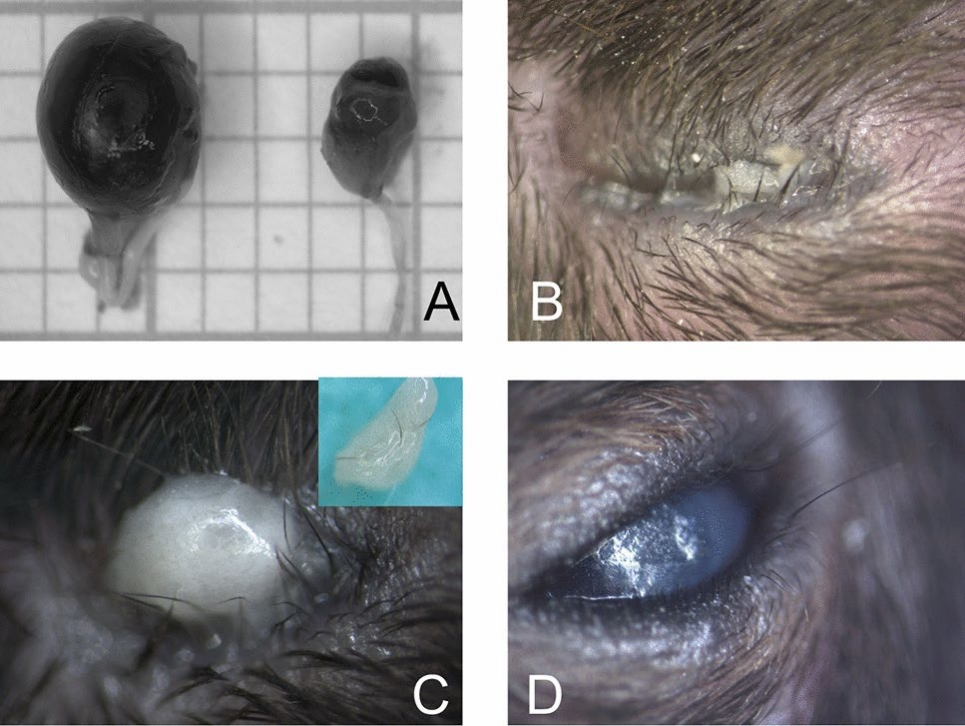
Representative images of microphthalmic eyes (**A**) Measurement of the eyeballs (same animal) per individuum on millimeter paper (**B**) Completely narrowed palpebral fissure and yellowish-mucous deposits on the lid margin (**C**) Missing part of the eyeball overlaid with a white-yellowish spherical deposit which, after removal, appeared as a completely viscous mass (inset). The mass was misinterpreted as opacification of the cornea (**D**) Narrowed palpebral fissures of a reduced eyeball whose cornea was completely opaque.

We recorded abnormalities in the lid margin, which were accompanied by crusting and swelling, punctured vibrissae and ocular discharge, as well as deposits of varying color and consistency in the corner of the eye and lid margin (Fig. 6 A-F). In cases in which whitish viscous secretions were released, active Meibomian glands could be detected by histologic evaluation accompanied by inflammatory cells infiltration at the lid margin (Fig. 6G). Eyes showing viscous secretion that adhered to the hair and lid margin, inflammatory cell infiltrates were detected in sections as well. Signs of infections ranged from swollen eyelids, injected conjunctiva or discharge of tear fluids. Of six individuals with suspected infection (Fig. 6 H–K), swab samples of the secretions were examined. Bacteria (*Staphylococcus (Staph.) aureus*, *Staph. xylosus* and not further classified *Streptococcus species)* were identified by culture and samples of affected eyes in three different individuals. Conjunctival hemorrhages were detected in a few individual cases, with the hemorrhage covering more than half of the eye (Fig. 6 L). Since 35 cases with underdeveloped eyes were recorded in our survey, we systematically examined these cases. The eyes were removed from the carcass and the globe diameters were measured (Fig. 7 A). In 27 cases, the right eye was small or missing. In 8 cases the left eye. Microphthalmic eyeballs are smaller (mean axial length 2.4 mm (right) vs. 3.0 mm (left), +/- SEM 0.7 mm, n = 12, not significant, p > 0.05) compared to healthy eyeballs. Smaller or missing eyes were associated with partially closed eyelids (mean eyelid fissure length 2.5 mm (left) +/- SEM 1.1 mm vs. 3.5 mm (right) +/- SEM 1.1 mm, n = 12, not significant, p > 0.05) and secretory fluids accumulating on the eye ocular surface or the eyelid margin (Fig. 7 B,C). In most cases with smaller eye appendices, opacities of the cornea were also detectable.

### Management and parameters

**Fig. 8:**
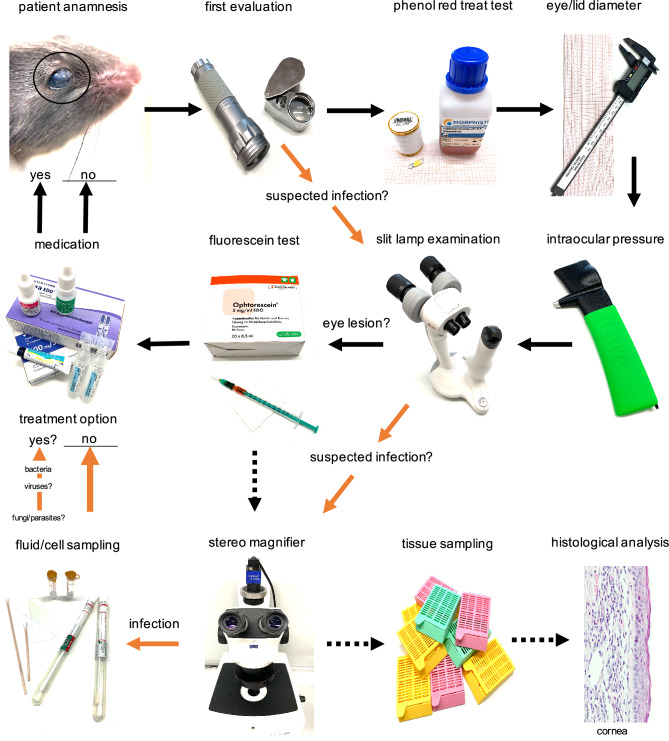
Proposed eye examination scheme for the routine procedure during ocular health evaluation in mice.

Based on the procedure we carried out for the systematic examination of mouse eyes in the context of herd management, we have summarized the procedure in a schematic diagram (Fig. 8). The measured value for tear production was reduced more frequently on the right eyes (mean 2.3 mm/15 s on the right side vs. 1.9 mm/15 s on the left side, n = 72, not significant, p > 0.05). There was a slight trend towards higher measured values in older animals (Supplementary Figure S2A). Measurement results of the intraocular pressure (IOP) (Supplementary Figure S2B) also showed elevated values on the right eyes (mean 15.4 mmHg on the right side vs. 11.5 mmHg on the left side, n = 29/27, not significant, p > 0.05) preferentially in cases of diffuse cornea opacity and/or fluorescein positive cornea lesions.

## Discussion

To ensure animals wellbeing, various parameters are used to measure or categorize pain, stress and distress in laboratory species as part of a research experiment^25^. Although criteria exist on which trained personnel can base stress assessments during routine animal inspections, comparatively little attention is given to the eye, eyelids, and the visual sensory apparatus, despite their relevance for sensory perception and behavior^28^. This may be due to the fact that some eye lesions in rodents are described in the literature as so-called “background lesions”^9^that should have little or no impact on the animal’s health and the reason that other senses like touch, olfactory or auditory can take over and compensate visual impairment^29,30^.

Experimental studies in visually impaired mouse models demonstrate that although animals may partially compensate for vision loss through other sensory modalities, such impairment is associated with altered exploratory behavior and stress-related responses, indicating that sensory compensation does not necessarily imply unimpaired welfare and unimpaired neuronal structures^31,32^. Precisely because of the background lesions and structural changes in the brain of visual impaired animals^30^, both the husbandry of the animals should be adapted with regard to their burden and their use in experiment should be taken into account.

In the current study we investigated to which extent staff working in laboratory animal facilities across three countries are involved in the diagnosis of ocular abnormalities with a strong focus on lesions of the anterior segment of the eye. The majority of participants have written instructions for the diagnosis whilst in parallel instructions for the management of ocular lesions are lacking. The observation that diagnostic measures are mainly initiated only after ocular alterations have become clinically apparent, while preventive strategies and systematic root-cause analyses are rarely applied, contrasts with current recommendations for refinement and burden reduction in laboratory animal science^33^. Therapeutic measures were only initiated in less than a half of the cases. The rejection of affected animals from the colony/breeding seems to be more important than treatment. From an ethical point of view, a diagnosis should come first, as death as a last resort is a reasonable option. This would be in line with the 3Rs model in which refinement is clearly part of ethical trial management^8^.

Only a few of the participants stated that they have the technical equipment to perform ophthalmic evaluation in-house. This illustrates how rarely diagnostic tools are used to screen for ocular lesions and thus they may go unrecognized in many cases. The use of fluorescein tests was indicated by up to 20% of the participants as a method which, in our estimation, should only be used in conjunction with a slit lamp with magnification, particularly in less severe cases. The fourth most common findings were wounds or injuries to the eyelids, which were selected by half of the participants. A high proportion of reported eyelid injuries suggests that *housing conditions*such as cage space allowance, social grouping and environmental complexity may influence general animal welfare. Experimental studies indicate that variations in space allocation and group size affect behavioral and physiological welfare measures in laboratory mice, including increased aggression and stress responses under certain conditions^34,35^. Moreover, cage density and cage environment parameters (e.g., ammonia levels) can alter environmental stressors that are known to influence health and behavior^36^. Although these studies do not specifically report ocular trauma, they support the notion that suboptimal housing is associated with compromised welfare, which may secondarily predispose animals to a range of lesions including those of the ocular adnexa. It is worth mentioning at this point that not all eye changes can be dismissed as “background lesions” in mice. Particular attention should be paid to changes of the corneal surface, which occurred in at least 28 cases in our survey. Similar to humans, the layers of the cornea become particularly sensitive to pain as fibers of the trigeminal nerve run through it, leaving an extensive network of free nerve endings that make damage to the cornea extremely painful^37,38^. Experimental mouse studies show that corneal injury activates nociceptive pathways and induces pain-related behavioral responses^37^, indicating that corneal lesions should be regarded as potentially painful conditions requiring prompt diagnosis and treatment with systemic analgetics and topical medication to prevent scar formation.

More than 43% of the survey participants were able to investigate infections disease throughout in-house bacteriology or virology. This pinpoints towards a strong connection of the monitoring of eye health and the detection of infectious agents by the facility staff. Our assumption is supported by the accumulation of symptoms such as swelling of the conjunctiva with conjunctivitis and inflammation of the eyelid margins, which accounted for 50—55% of all responses being the third most common observations in mice in our survey. However, bearing in mind that most of the agents listed in FELASA recommendation^39^ will not cause primary ocular manifestations, despite some bacteria, e.g., *Pasteurella sp.,* causing this in laboratory or pet mice^40,41^we argue that mice housed in specified-pathogen-free laboratory facilities are very well protected from acquired infectious diseases. However, acquired primary lesions due to environmental factors may predispose them to the colonization of opportunistic agents. Indeed, we could identify three bacteria species by swab sampling which are known to be commensals of the skin and can lead to secondary eye manifestations especially in cases of immune suppression^42,43^..

The second most common veterinary clinical observation is microphthalmia. That lesion is associated with a genetic predisposition predominantly in inbred C57BL/6 mice^7,13^. As described by Burkholder et al*.* microphthalmia leads to impaired drainage of tear fluids^7^which can predispose for infectious diseases of opportunistic agents and may require treatment of concomitant conjunctivitis^7,13^. In our investigation, we identified 35 cases of microphthalmic eyes without a predisposition for one side of the body. Microphthalmia was associated with cornea opacity in almost all cases.

Developmental ocular anomalies such as microphthalmia may also affect the establishment and organization of corneal innervation. In a well-characterized murine “small eye” model, heterozygous *Pax6*mutant mice, Leiper et al. demonstrated a markedly altered pattern and reduced density of corneal sensory nerve fibers, indicating that normal corneal innervation critically depends on intact eye development^44^. These findings suggest that microphthalmic eyes may exhibit not only structural abnormalities but also impaired sensory innervation of the cornea. Although direct evidence linking impaired corneal innervation to altered pain sensitivity in mice showing microphthalmic eyes is currently limited, these developmental alterations should be considered in the management of mouse colonies and should be euthanized for animal welfare reasons. Further, since a genetic component in these types of lesions is obvious or has been proven^9^, such animals are not suitable for breeding or experimentation. In our survey, lens opacities (cataract formation) were recorded up to 78.59% times. That lesion seems to be a common background lesion in mice and are due to genetics, ageing or experimental procedures^9,19,45^. Lens opacities come in different forms (anterior/posterior or pre-mature/mature), but they all have in common that the passage of light is reduced and thus, as in humans, visual impairment develops over time. It should be stated here, opacities can be misinterpreted by diffuse clouding of the cornea as those resemble cataract formation in mice^9^. In our investigation of 142 ocular lesions, 13 out of 18 cases of lens opacity required slit lamp examination for precise diagnosis of early cataract formation.

Without appropriate ophthalmic diagnostic tools, particularly anterior segment biomicroscopy using a slit beam, gross visual inspection of the mouse eye lacks optical sectioning and retroillumination and therefore does not allow reliable localization of opacities to the cornea versus the lens. In standardized ocular phenotyping of laboratory mice, slit-lamp biomicroscopy is explicitly used to differentiate corneal stromal haze or deposits from lenticular abnormalities, as direct inspection and biomicroscopic examination can yield substantially different impressions of the same lesion. Large-scale phenotyping studies in C57BL/6-derived strains demonstrate that accurate classification of anterior segment pathology requires magnified slit-beam examination and photographic documentation, often in combination with complementary methods such as optical coherence tomography or histology, to avoid misinterpretation of corneal opacities as cataracts^45,46^. We could identify 18 cases showing cataract formation in some cases associated with e.g*.,*iris deformation, cornea-lens adhesion which were diagnosed by slit lamp examination or by histological evaluation. We assume that lens lesions are overrated in routine rodent colony management, since based on the result of our survey, slit lamps were rarely used. Although lens opacity itself does not cause pain, visual impairment can cause discomfort to an animal, which is assumed to be difficult to measure in mice since existing evaluation forms primarily focus on pain responses^22^. Since cataract-induced visual impairment develops over time and can be compensated for by other sensory systems, the impact of lens opacities on animal welfare is not clear. Lens opacities themselves are not considered painful per se but secondary complications such as impaired aqueous humour outflow and glaucoma may lead to pain and distress. The production and absorption of aqueous humour is comparable to the situation in humans^47^, which allows for a direct interpretation of the consequences of increased IOP values, which are associated with ischemic damage and pain^48,49^. IOP measurement has been characterized in genetically diverse mouse strains demonstrating both baseline differences between strains and circadian effects on IOP that are relevant for phenotyping^50^. Measurement of intraocular pressure is therefore an established method in mouse models to detect secondary ocular pathology and should be considered a refinement measure in routine ophthalmic assessment.

Our survey showed that IOP measurement would only be available in the rarest of cases. Interestingly, in our own examinations, the number of mice showing cataract formation is low compared to overall cornea opacities diagnosed by us. We therefore hypothesize, that without adequate equipment and training misdiagnosis might be the explanation for those high numbers of cataracts given in the survey. In our systematic approach, as described in Fig. 8, measurements of tear film production rate and intraocular pressure showed deviations in the respective diseased eyes, which speaks for a sensible use of the methods in diagnostics. In view of the simplified applicability and the greater benefit for the diagnosis of ocular changes, we recommend the use of IOP measurement in routine ophthalmic investigation of the cornea and anterior chamber in mice. Further, we could show by histological investigation, cornea thickening is associated with neovascularization and in severe cases with immune cell infiltration and hypopyon. From an animal welfare perspective, it’s ethically mandatory to diagnose eye lesions adequately to prevent any further burden arising for the animal. A mere assumption that eye changes in mice are due to genetic causes might cause insufficient or inappropriate treatment. Rather, ocular alterations and their adequate diagnosis in the day-to-day operation of a laboratory animal facility must be given weight in the assessment of animal welfare. From our own experience at our facility, the number of cases depends on whether or not the animals are examined by trained personnel. The number of unrecognized eye lesions may therefore be significantly higher than previously assumed. In 26 cases, lesions could be detected in the other eye that were not recognized during the first examination by untrained persons without technical aids.

In our survey, roughly 70% of the participants reported that the causes of eye changes were not systematically investigated. This suggests that such findings are often seen as unimportant, with underlying causes inadequately addressed, and the individual animal’s stress largely overlooked—falling short of ethical animal welfare standards. Although professional guidelines recommend including the eyes in health assessments, there are still no established criteria for distinguishing between painful and non-painful eye lesions.

Healthy eyes are crucial for animals to perform essential functions such as navigation, recognizing conspecifics, and species conservation, so any deviation from normal physiological conditions must be considered a burden and ethically evaluated when using them for breeding or experiments. Therefore, routine eye examinations and clear stress criteria are necessary in laboratory animal science to properly assess eye changes, their impact on welfare, to support the use of animals with normal eyes in neurological research, and to support ethical decision-making.

## Methods

### Questionnaire

A cross-sectional survey (convenience sampling), was conducted among laboratory animal professionals in Germany, Switzerland, and Austria. The survey was developed using SurveyMonkey (Momentive Europe UC, Dublin, Ireland). Ethical approval for the study was obtained from the Animal Welfare Committee of the Core Facility Animal Models (Medical Faculty of the Ludwig-Maximilians-Universität München. By actively participating in the online survey, respondents confirmed their consent to the anonymous evaluation and publication of the data.

Twenty questions were asked about demographics, institution, and management of eye abnormalities. In addition to quantitative data, participants were asked to provide free-text feedback regarding the circumstances under which further diagnostics are performed, eye abnormalities diagnosed, and potential factors contributing to these abnormalities.

Pre-assessment was conducted with three individuals representing key stakeholder groups (scientist, caretaker, named veterinarian) using cognitive interviewing. Subsequently, a pilot study was carried out involving three additional participants from relevant professional backgrounds.

A translated version of the questionnaire is provided in Supplementary Table S1. An overview of the item structure, filter navigation, and respective number of responses are shown in Supplementary Table S2.

The survey was accessible online from March 20th to May 5th, 2025. The access link was disseminated via interest groups and professional associations involved in laboratory animal care and use. Only individuals currently responsible for housing and care of laboratory mice were eligible to participate. Data collection, storage, and processing were conducted in accordance with German data protection regulations. Participants were informed about the objectives and methodology of the study, and informed consent was required prior to survey initiation.

Participation was voluntary and anonymous; no IP addresses or other personal identifiers were recorded. Survey responses were exported from SurveyMonkey and processed using Microsoft Excel. Incomplete responses (surveys not submitted or with > 30% missing data) were excluded from analysis. Quantitative data were analyzed descriptively and qualitative data were assessed using inductive evaluation. The design and report for this study were performed in accordance with relevant guidelines and regulations as well as standards for reporting of survey studies (CROSS)^51^.

### Animals

The housing of laboratory mice was in accordance with European (RL 2010/63EU) and German animal welfare legislations (5.1–231 5682/LMU/BMC/CAM) as previously reported^52^. The study was conducted in accordance with the ARRIVE guidelines. All protocols and procedures involving animals or materials complied with the applicable local regulations and the recommendations and guidelines of the Core Facility Animal Models (Medical Faculty of the Ludwig-Maximilians-Universität München). A total of 142 animals of both sexes (age range 1–31 months) were included in the evaluation. 127 animals either heterozygous or homozygous for at least one genetic modification (GMO) and 15 wildtype (WT) animals were analyzed. Mice were either bred in-house or purchased from commercial breeders and were not used in any animal testing projects at the time of the veterinary assessment or previously.

### Ophthalmic assessments

142 animals were evaluated after indexing of “any eye abnormalities” by animal caretakers in the animal husbandry database (tick@lab, a-tune software AG, Darmstadt, Germany) using terms like e.g., cataract, cloudy eye, blind, missing eye. Eye examination was performed by a trained veterinarian and an experienced bachelor of laboratory animal science. We took magnifying pictures of all mice investigated for pre-diagnosis before sending over to the veterinarian.

### Initial eye examination

Routine examination consists of an inspection of the eyelids, surrounding area and eye ocular surface. A jeweler’s magnifier (EEEkit, Model 1, Fremont, CA, USA) was mounted on a smartphone (iPhone 10, Apple Inc., Cupertino, CA, USA) for digital image acquisition and appropriate illumination. Mice were gently placed on cage lids during the approximately 30 s of examination and, if necessary, gently hold by the scruff of the neck. Based on this initial assessment, the subsequent tests were selected to obtain a comprehensive picture.

### Tear secretion

Mice were gently fixated by hand and self-made phenol red thread test (PRTT)^53^method was applied adapted from Puentes at el^54^. (phenol red 13,664, Morphisto, Offenbach am Main, Germany). The device was placed in the nasal cantus of the right and left eye, after 15 s, the wetted length of the thread, indicated by a color change from yellow to red was determined in millimeters (2613, PVP Papierverarbeitung GmbH, Penig, Germany).

### Intraocular pressure

Directly after the tear secretion test, mice were euthanized by cervical dislocation, and intraocular pressure (IOP)^55,56^ was determined for both eyes using the TONOVET tonometer (Icare, Eickemeyer, Appenzell, Switzerland) calibrated for mice. The mean value IOP per eye was calculated by the TONOVET software after deleting the highest and lowest measured values from six consecutive individual measurements.

### Anterior segment inspection

Examination of the cornea, anterior chamber, iris and lens was performed using a hand-held slit lamp^57^ (Kowa SL19, Tokyo, Japan) at 10 × or 16 × magnification. Digital images were taken using a smartphone (iPhone 10, Apple Inc. Cupertino, USA) mounted on the binocular.

To test for corneal erosion or ulcer, the fluorescein test (Opthoresicein 5 mg/mL EDO, CP-Pharma, Burgdorf, Germany) was performed^58^ The coloring was examined under blue light by using the slit lamp. Images were taken using a stereomicroscope (Stemi 508, Carl Zeiss AG, Jena, Germany) equipped with a digital camera (Axiocam 105, Carl Zeiss AG, Jena, Germany) for documentation.

### Eyelid diameter measurement

Interpalpebral fissure width was measured in cases of smaller appearing eyeballs by the use of a digital electronic caliper (FST, Heidelberg, Germany)^56^. Further, to measure eyeball dimensions, eyeballs were carefully dissected from the carcass and placed on micrometer paper sheets (2613, PVP Papierverarbeitung GmbH, Penig, Germany) for image acquisition and axial length determination.

### Swab sampling

In case an underlying infectious disease (e.g., eye discharge, swelling of eyelids, redness of conjunctiva) was suspected, swab samples for PCR (Snooplex, FastPrep Kit, GVG Genetic monitoring, Leipzig, Germany) or culture (dry swab 1,020,002, Heinz Herenz, Hamburg, Germany) were taken from the lower lid margin and conjunctiva. The samples for bacteriological investigation were collected and stored in accordance with the manufacturer’s instructions and sent to an external laboratory for further analysis. The samples were examined for the presence of frequently identified pathogens (*Pasteurella/Rodentibacter sp., Staphylococcus sp., Streptococcus sp*^59,60^. Testing for specific germs was carried out by cultivating bacteria on agar followed by PCR analysis to confirm bacteria species.

### Histology

Animals that underwent histological examination were euthanized by cervical dislocation. Samples (eyeballs, eyelids or adjoined tissue) were formalin-fixed (Roti Histofix, Carl Roth GmbH + Co. KG, Karlsruhe, Germany) and paraffin-embedded. Sectioning was performed on microtome (HM 355S, Microm, Leica Microsystems GmbH, Wetzlar, Germany) adjusted to 5 µm section thickness. Sections were mounted on glass slides (K016, Diagonal GmbH & Co. KG, Münster, Germany) and stained with hematoxylin and eosin (H&E) (12,156, Morphisto GmbH, Offenbach am Main, Germany). Images were acquired using a Axiovert 5 microscope connected to a Axiocam 205 (Carl Zeiss AG, Jena, Germany) camera system. Image analysis was done by the use of the ZEN software (version 3.11, Carl Zeiss AG, Jena, Germany).

### Statistics

Statistics were conducted using Prism GraphPad 5.04 (GraphPad Software, San Diego, CA, USA). Student’s *t*-test was applied for parametric data after confirmation of normal distribution by Kolmogorov–Smirnov test. No multiple comparisons or statistical corrections were made. The statistical calculation was performed based on the actual number of cases; the sample size was not calculated in advance (power analysis), as the number of cases that would occur during the study period was unknown. Data are shown as mean and SEM values if not stated otherwise. The significance level was set to *α* < 0.05.

## Supplementary Information

Supplementary Information 1.

Supplementary Information 2.

Supplementary Information 3.

Supplementary Information 4.

## Data Availability

All data generated or analyzed during this study are included in this published article and its supplementary information files.
