# Supplementary material for: Prevalence and management practices of ophthalmic lesions in laboratory mice
Source: Sci Rep. 2026 Mar 11;16:8732. doi: 10.1038/s41598-026-43181-9 (PMC12979715; doi:10.1038/s41598-026-43181-9)
Supplement: Supplementary file 1 — Supplementary Information 1. [file 41598_2026_43181_MOESM1_ESM.pdf]

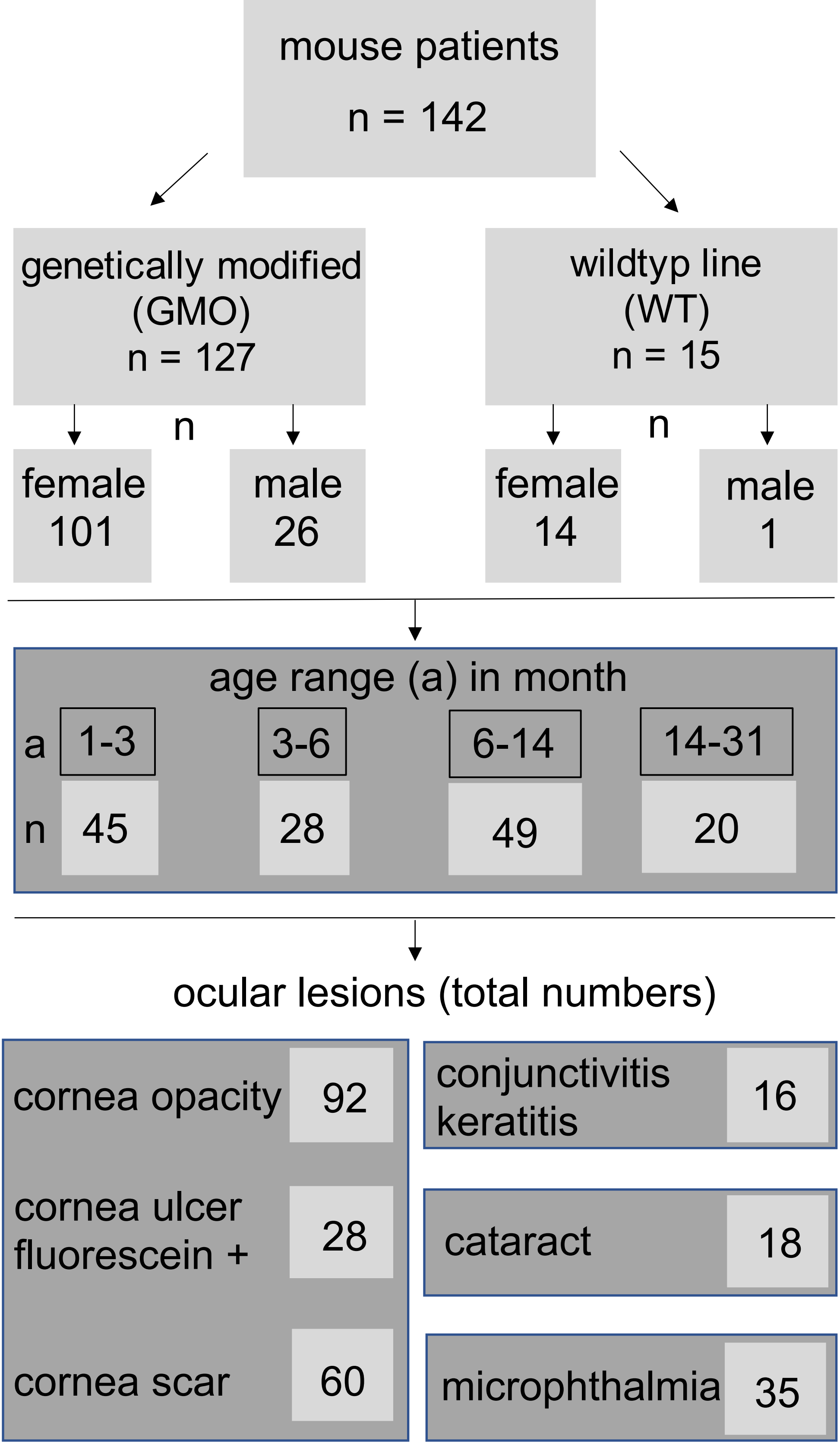
mouse patients
n = 142
genetically modified
(GMO)
n = 127
wildtyp line
(WT)
n = 15
n
female
101
male
26
n
female
14
male
1
age range (a) in month
a
1-3
3-6
6-14
14-31
n
45
28
49
20
ocular lesions (total numbers)
cornea opacity
92
cornea ulcer
fluorescein +
28
cornea scar
60
conjunctivitis
keratitis
16
cataract
18
microphthalmia
35
