# Supplementary figures and images for: Prevalence and management practices of ophthalmic lesions in laboratory mice
Source: Sci Rep. 2026 Mar 11;16:8732. doi: 10.1038/s41598-026-43181-9 (PMC12979715; doi:10.1038/s41598-026-43181-9)

**A**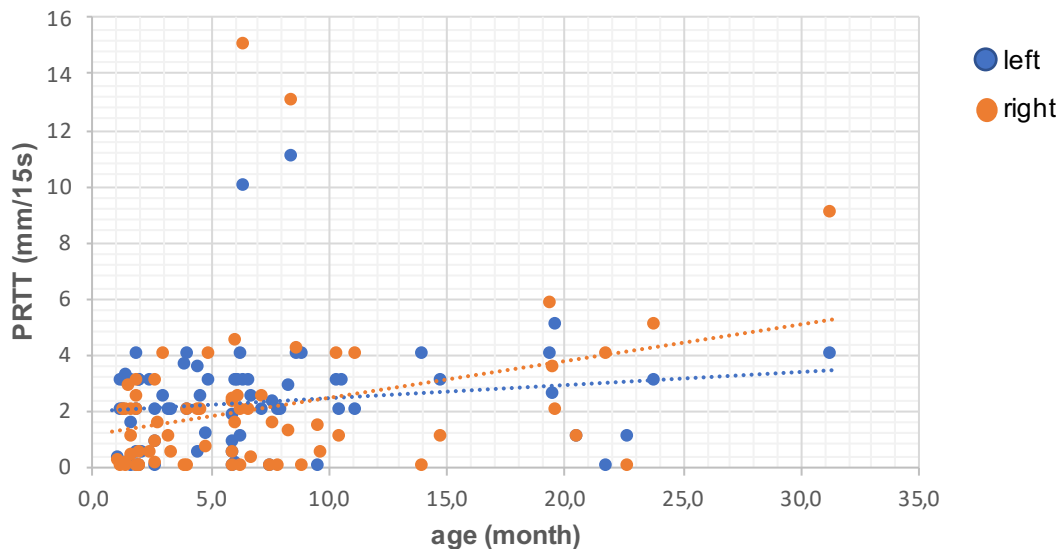**B**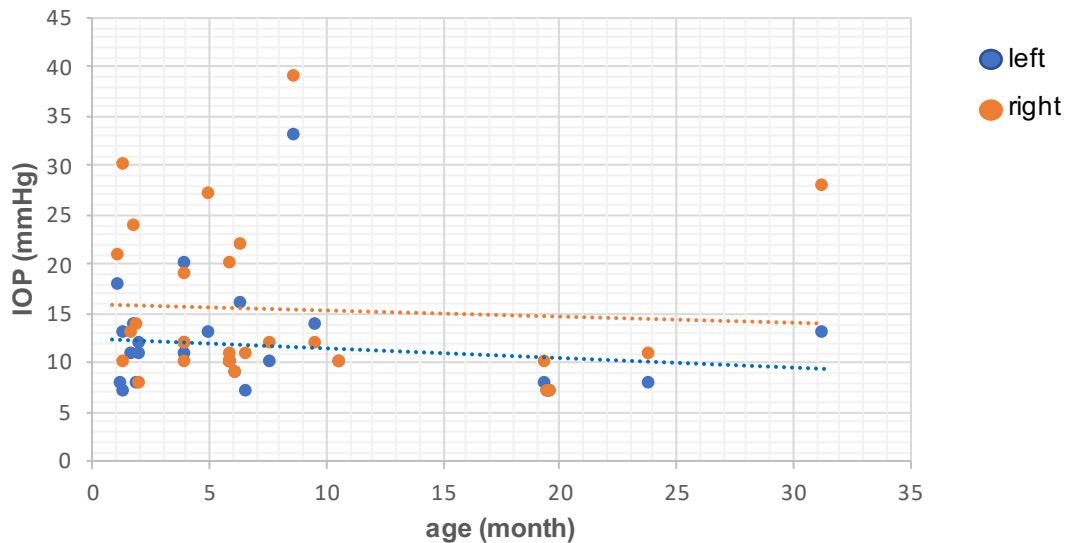

Supplement: Supplementary file 2 — Supplementary Information 2. [file 41598_2026_43181_MOESM2_ESM.pdf]

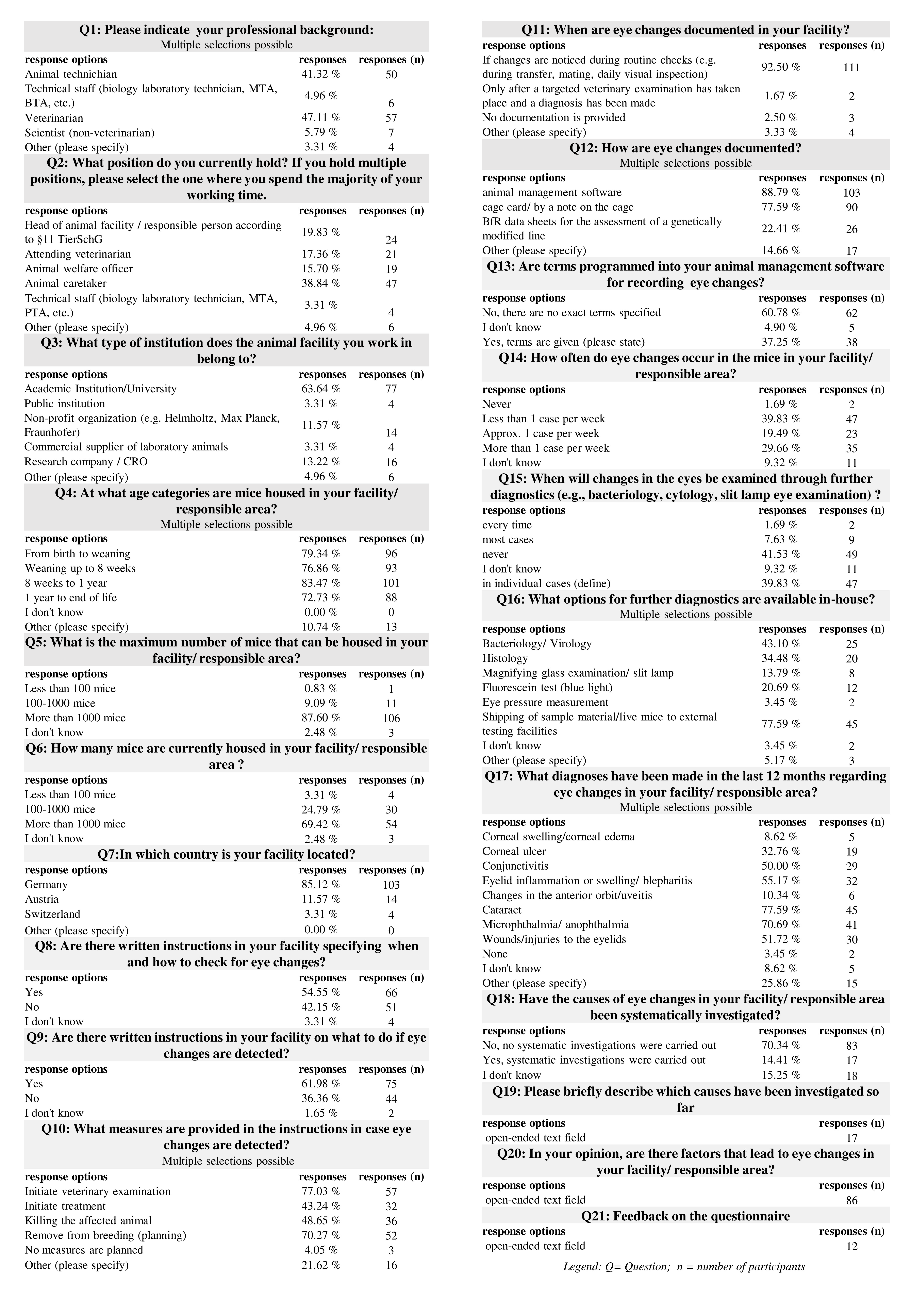

Supplement: Supplementary file 3 — Supplementary Information 3. [file 41598_2026_43181_MOESM3_ESM.tif]
