# Supplementary material for: Prevalence and management practices of ophthalmic lesions in laboratory mice
Source: Sci Rep. 2026 Mar 11;16:8732. doi: 10.1038/s41598-026-43181-9 (PMC12979715; doi:10.1038/s41598-026-43181-9)
Supplement: Supplementary file 4 — Supplementary Information 4. [file 41598_2026_43181_MOESM4_ESM.pdf]

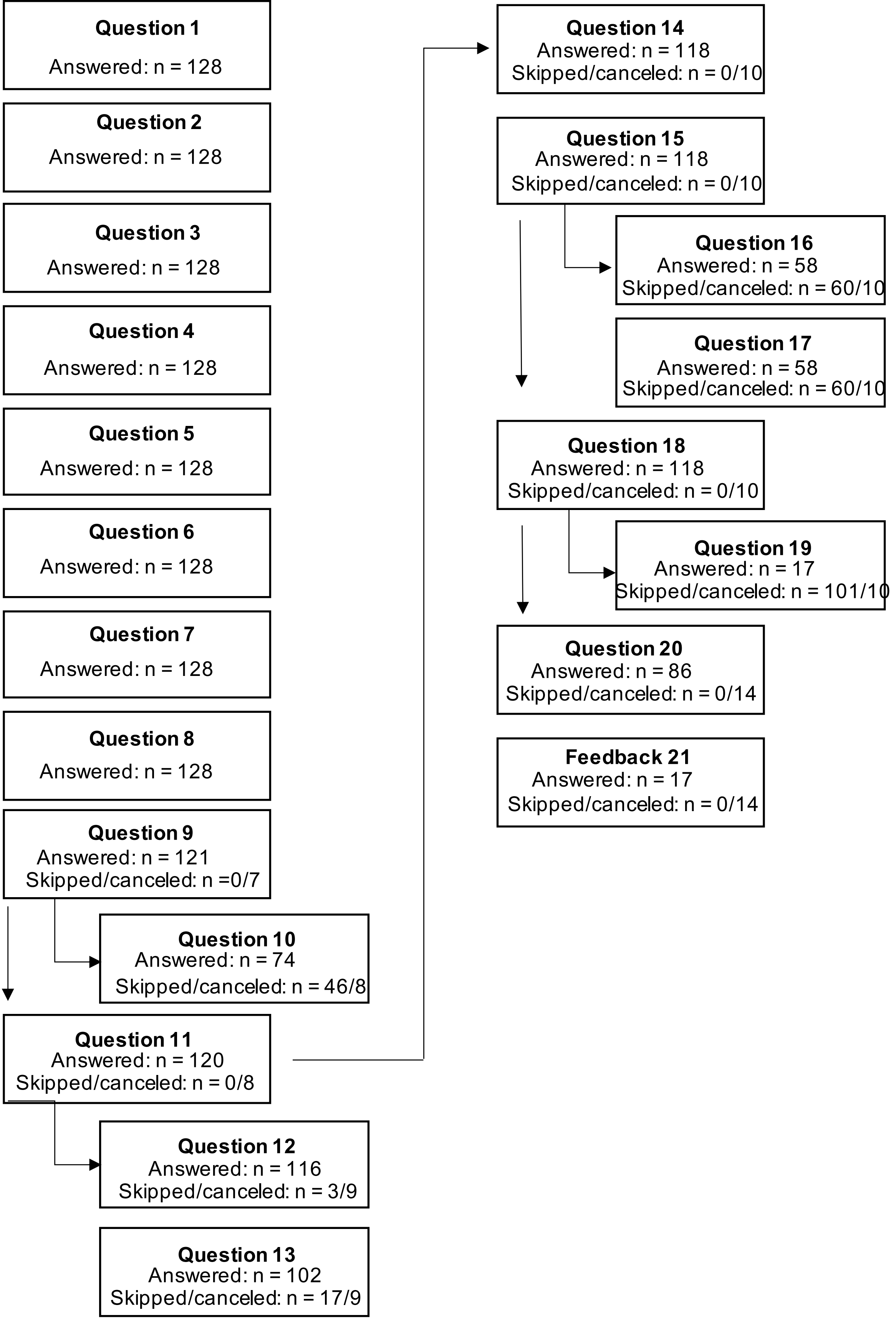

Question 1
Answered: n = 128
Question 2
Answered: n = 128
Question 3
Answered: n = 128
Question 4
Answered: n = 128
Question 5
Answered: n = 128
Question 6
Answered: n = 128
Question 7
Answered: n = 128
Question 8
Answered: n = 128
Question 9
Answered: n = 121
Skipped/canceled: n =0/7
Question 10
Answered: n = 74
Skipped/canceled: n = 46/8
Question 11
Answered: n = 120
Skipped/canceled: n = 0/8
Question 12
Answered: n = 116
Skipped/canceled: n = 3/9
Question 13
Answered: n = 102
Skipped/canceled: n = 17/9
Question 14
Answered: n = 118
Skipped/canceled: n = 0/10
Question 15
Answered: n = 118
Skipped/canceled: n = 0/10
Question 16
Answered: n = 58
Skipped/canceled: n = 60/10
Question 17
Answered: n = 58
Skipped/canceled: n = 60/10
Question 18
Answered: n = 118
Skipped/canceled: n = 0/10
Question 19
Answered: n = 17
Skipped/canceled: n = 101/10
Question 20
Answered: n = 86
Skipped/canceled: n = 0/14
Feedback 21
Answered: n = 17
Skipped/canceled: n = 0/14
